# Supplementary material for: Chromothripsis during telomere crisis is independent of NHEJ, and consistent with a replicative origin
Source: Genome Res. 2019 May;29(5):737–49. doi: 10.1101/gr.240705.118 (PMC6499312; doi:10.1101/gr.240705.118)
Supplement: Supplemental Material [file supp_gr.240705.118_Supplemental_file_1.zip › contigs/annotated_contigs/DB55/contig.2.DB55_length_717_mean_cov_5.49232914923.docx]

**DB55_length_717_mean_cov_5.49232914923**

```
CATAAATATGAATATTTGTTCCTTTGTAATCTGGCTCTTCCTGTGATAAGATGCCACCAATATTTGGGTAGAATGCTGGGGAGAAGTTA
 >chr1:245354408-245354598 + E=2e-99 p=2e-03
CCTCCCAACATTGAAAAGAGTAAAATCCCAGCACTTTGGGAGGCCGAGGCGGGCGGATTACCTGAGGTCAGGAGTTCGAGACCAGCCTG

GCCAACATGGTG|GCCAACATGG|TGTTGTTGTTGTTTTGAGACAGAGTCTCACTCTGTTGCCCTGGCTGGAATGCAGCAGCACAATCT
                       >chr1:244910907-244911398 + E=2e-261
CTGCTCACTGCAACCTCCATCTCCCGGGTTCAGTTCAAGTGATTATCATGCCTCAGCCTCCCAAGTAGCTGGGATTACAGGCGCACGCC

ACCATGCCCGGCTAATTTTTGTATTTTTAGTAGAGATGGGGTTTCACCACGTGGACCAAGTTGGTCTGGAACTCCTGACCTCAAGAGAT

CTGGCCGCCATGGGCTTCCAAAGTGCTGGGATTACAGGGATGAGCCACCGTGCACAGCCTAAATTTTTTTGTGGTGGTTTTTATCTTTT

TAATTTTTTTTATTTTTGTTTTTTCTTTTTCTTATTTTTTATTTTTTCTTTCTTCTTTGTTTTTCTCTTTCAGTTTTATTTCTTTATTT

TAGTGGTTTTTTTTTCACAAGTAGGGTTTGTTTTGTTTTGTTTTTTTGAGATTGAGTTTGGCTCTGTTG|GCCCGGTTTGAGGGCATTG

GCTCTATA
```
